# Insight Into the Roles of Non-coding RNA in Bronchopulmonary Dysplasia

**DOI:** 10.3389/fmed.2021.761724

**Published:** 2021-11-05

**Authors:** Yufeng Xi, Yujia Wang

**Affiliations:** ^1^Department of Neonatology, Chengdu Women's and Children's Central Hospital, School of Medicine, University of Electronic Science and Technology of China, Chengdu, China; ^2^Department of Dermatology, State Key Laboratory of Biotherapy, West China Hospital, Sichuan University, Chengdu, China

**Keywords:** bronchopulmonary dysplasia, premature infants, noncoding RNA, microRNA, long non-coding RNA

## Abstract

Bronchopulmonary dysplasia (BPD) is a chronic lung disease most commonly occurring in premature infants, and its pathological manifestations are alveolar hypoplasia and dysregulation of pulmonary vasculature development. The effective treatment for BPD has not yet been established. Non-coding RNAs, including microRNAs and long non-coding RNAs do not encode proteins, but can perform its biological functions at the RNA level. Non-coding RNAs play an important role in the incidence and development of BPD by regulating the expression of genes related to proliferation, apoptosis, angiogenesis, inflammation and other cell activities of alveolar epithelial cells and vascular endothelial cells. Here we summarize the role of non-coding RNAs in BPD, which provides possible molecular marker and therapeutic target for the diagnosis and treatment of BPD.

## Introduction

Bronchopulmonary dysplasia (BPD) (1–4) is an important cause of respiratory illness in preterm newborns that results in significant morbidity and mortality. BPD was first discribed by Northway in 1967, which was caused by oxygen supply and mechanical ventilation in premature infants with severe respiratory distress syndrome (5). The “new” BPD pathology is characterized by arrested alveolar-capillary development with larger, simplified alveoli, increased interstitial fibrosis, and abnormal pulmonary vasculature with decreased branching (1, 6–8). Clinical epidemiological studies (9) show that BPD occurs mostly in premature infants, and the younger the gestational age and the lower the birth weight, the higher the incidence and severity of BPD. Infants with BW < 1,250 g accounted for 97% of BPD cases. BPD has an important impact on the respiratory and nervous system, which seriously affects the quality of life of children, and at the same time, it will cause heavy economic burden on the family and society (10). Despite prenatal usage of glucocorticoid (11), pulmonary surfactant (12), moderate ventilation strategy (13) and other methods improve the survival rate and decrease the incidence rate of BPD in premature infants, but BPD remains an important cause of death for very low birth weight infants. Current studies suggest that BPD may be caused by a combination of multiple factors including premature birth (14, 15), fetal growth restriction (16), mechanical ventilation (17), oxygen toxicity (18), infection (19), inflammation (20), patent ductus arteriosus (PDA) (21), genes and genetic susceptibility (22) and so on. So far, the etiology and pathogenesis of BPD are not yet clear, thus there is no specific treatment (23).

Non-coding ribonucleic acid (RNA) refers to RNA that does not encode proteins (24). Non-coding RNA include ribosomal RNA (rRNA) (25), transfer RNA (tRNA) (26), small nuclear RNA (snRNA) (27), small nucleolar RNA (snoRNA) (28), small interfering RNA (siRNA) (29), circular RNA (circRNA) (30), microRNA(miRNA) (31), and long non-coding RNA(LncRNA) (32) with known functions, as well as RNA with unknown functions. The common feature of these RNAs is that they can be transcribed from the genome, but not translated into proteins. However, these non-coding RNAs play an important role in gene expression and disease progression, and they can regulate gene expression at transcriptional or post transcriptional levels. In recent years, molecular biology research has found that non-coding RNAs play an important role in the incidence and development of lung diseases (33), such as lung cancer (34), chronic obstructive pulmonary disease (COPD) (35), asthma (36), respiratory distress syndrome (RDS) (37) and BPD, which provides a new idea for the treatment of diseases (38).

In this review, we summarize the role of non-coding RNAs in BPD, in order to provide possible molecular marker and therapeutic target for the diagnosis and treatment of BPD.

## The Roles of Non-coding RNA in BPD

### MiRNA

MicroRNA (miRNA) is a class of endogenous non-coding single stranded RNA with a length of about 20–24 nucleotides (39). It regulates gene expression by inhibiting translation or mediating the degradation of target mRNA at the post transcriptional level (40). Each miRNA can have multiple target genes, and several miRNAs can regulate the same gene at the same time. Studies have shown that miRNAs can regulate 30% of all genes in the human genome and play an important role in various biological processes, including cell proliferation, differentiation and apoptosis, metabolism, tissue damage and repair, biological development and tumor occurrence and development (41). More and more research suggest miRNA has been involved the incidence and development of BPD, and the summary is as follows.

MiR-17~92 clusters are needed to be appropriately expressed in normal lung growth and development, and changes in miR-17~92 clusters have also been reported in other lung diseases (42, 43). Data from severe BPD murine model suggested that the expression of miR-17~92 cluster in lung tissue is downregulated (44), meanwhile, autopsy tissue data suggested that the expression of miR17~92 is lower in BPD lung tissue than that in normal lung tissue, and is inversely correlated with promoter methylation and DNA methyltransferase expression. In addition, miR-17 and miR-19b expression was decreased in plasma sample in infants with BPD (45). MiR-17~92 clusters have been shown to regulate transforming growth factor-β(TGF-β), matrix metalloproteinases (MMPs), and Collagen III alpha 1 (COL3A1). Alter of matrix composition will decrease alveolarization and pulmonary function as observed in infants with severe BPD and in our murine model.

In the mouse model of BPD, hyperoxia exposure was associated with increased S-nitrosoglutathione (GSNO) catabolism by increasing the expression of GSNOR in the lungs. It is observed that the expression of miR-342-3p is decreased in mice exposed to hyperoxia. One of the mechanisms of upregulation of GSNOR protein expression induced by hyperoxia is *via* post-transcriptional regulation of miR-342-3p. It has been predicted that this mechanism may be related to other pulmonary toxicities associated with hyperoxia exposure (46).

Previous studies have found that abnormal remodeling of extracellular matrix (ECM) is related to the pathogenesis of BPD (47). Fibronectin 1 (FN1) as a major component of ECM, FN1 also has angiogenesis characteristics and can interact directly and indirectly with other key regulatory factors of ECM, such as TGF-β1 and VEGF (48, 49). In addition, FN1 is considered to be caused by inflammation (50, 51), and many studies have shown that inflammation is associated with an increase in severe BPD (52, 53). The expression of miR-206 in BPD mice tissues and BPD patient tissues is lower than that in normal tissues. Overexpression of miR-206 induced cell apoptosis, reduced cell proliferation, migration and adhesion. And FN1 is the direct target of miR-206. In BPD mice and BPD patients, the expression level of fibronectin 1 is increased. Downregulation of miR-206 increased the expression level of FN1, which played a role in ECM remodeling and contributed to BPD (54).

MiR-29a was significantly increased in the BPD mice lung tissue, and it has been proved direct miR-29a target were further validated using bronchoalveolar stem cells (55).

Hu Y et al., (56) found that down-regulation of miR-29a can potentially increase GRB2-associated-binding protein 1 (GAB1) expression, reducing cell apoptosis and stimulating proliferation, ultimately retarding the development of BPD in mice. Analysis of plasma in preterm infants with subsequent BPD showed that miR-29b decreased and was negatively correlated with the severity of BPD. The combination therapy of miR-29b with AAV9 as a carrier may hopefully restore normal lung structure in preterm infants with severe BPD (57). Suppression of miR-29b expression is related to the increasing of transforming growth factor-β (TGF-β), Smad2/3 protein levels, and all of which lead to extracellular matrix (ECM) deposition.

Delta-like 4 (DLL4), a membrane-bound ligand belonging to the Notch signaling family, plays a fundamental role in vascular development and angiogenesis (58). miR-30a regulates endothelial tip cell formation and arteriolar branching by downregulating Dll4 (delta like ligand 4) expression (59). In clinical studies, miR-30a was downregulated in preterm infants with BPD (60, 61). These studies indicate that decreased miR-30a expression may be associated with impaired lung development in neonates with BPD. Analysis of the pulmonary transcriptome revealed that miR-30a target angiogenesis-related genes, namely the mechanism is upregulation of miR-30a inhibiting the expression of Dll4 and subsequent downregulation of angiogenesis (61). Another study (62) shows that higher miR-30a expression through hypoxia inducible factor-1α (HIF-1α) decreases Snai1 expression in females and attenuates injury in the developing lung.

Hyperoxic acute lung injury (HALI) is a key factor in the pathogenesis of BPD. It has been found a significant increase in the level of miR-34a in BPD mice lung tissue. In the model of BPD mice, the absence or inhibition of miR-34a improved BPD-related pulmonary hypertension (PAH). Meanwhile, the use of angiopoietin-1 (one of the downstream targets of miR-34a) can improve the pulmonary phenotypes of PAH and BPD. In addition, it has been reported an increase in miR-34a level in type 2 alveolar epithelial cells in neonates with BPD. It suggested that drug-induced miR-34a inhibition may be a therapeutic option for BPD prevention and treatment (63). A study by Ruiz-Camp et al., (64) suggest that miR-34a in platelet-derived growth factor receptor α positive (PDGFRα+) cells contribute to aberrant lung alveolarization, but global loss of miR-34a partially restores lung alveolarization in experimental BPD.

The expression level of miR-489 in the lung tissue of BPD neonates is lower than that of normal neonates, which is related to increases in its conserved target genes insulin-like growth factor-1 (IGF-1) and tenascin C (Tnc) (65).

Glycoprotein non-metastatic melanoma protein B (GPNMB) is a transmembrane protein, which plays an important role in angiogenesis and tissue repair via inducing autophagy (66, 67). And it has been identified as one of the targets of miR-150 (68). The expression of miR-150 decreased in hyperoxia exposure neonatal rat lung tissues. And soluble GPNMB promoted angiogenesis in the hyperoxic neonatal mice lung tissues *via* miR-150 regulation. GPNMB played an important role in angiogenesis during hyperoxia injury and GPNMB therapy can provide a new method to reduce the pathological complications of BPD (69).

Fibroblast growth factor 10 (FGF10) is an important regulator of branching morphogenesis in the early stage of lung development, which is a potential marker for predicting the development of BPD in premature neonates (70, 71). FGF10 has been identified as a target gene for miR-421. The expression of miR-421 was significantly increased in BPD mice lung tissues while the expression of FGF10 is decreased. The upregulation of miR-421 and the silence of FGF10 aggravated the inflammatory response of lung tissue and promoted the apoptosis of Type II alveolar epithelial cell line MLE-12. These changes can be reversed by lowering the expression of miR-421. Inhibiting the expression of miR-421 can be helpful to the development of BPD in mice by upregulating the expression of FGF10. MiR-421 might be possible target for the prevention and treatment of BPD (72).

Adrenomedullin (ADM) (73), an important regulator of oxidative damage, has been reported that it can protect the premature infants with BPD. However, the pathogenesis of ADM regulating BPD is not clear. The study (74) finds that the expression of miRNA-574-3p in blood of premature infants with BPD was significantly lower than that of premature infants without BPD. Luciferase reporter gene analysis showed that ADM is the target gene of miR-574-3p. The increased expression of ADM regulated by miR-574-3p may protect premature infants with BPD and provide new ideas for the prevention and treatment of BPD. However, we need more intervention study to identify the idea.

It has been reported that the expression level of miR-876-3p is lower in BPD mice lung tissues and Human Bronchial Epithelial (NHBE) cells than that in normal tissues and cells. For increased proteobacteria in the airway microbiome is related to BPD, it has been reported a new model combining proteobacterial LPS and hyperoxia exposure. Compared with hyperoxia alone, the add of LPS can significantly reduce the expression of miR-876-3p in NHBE cells, which indicated that the change of microbial community can inhibit the potential mechanism of miR-876-3p (75).

Let-7, as an important family of Mir family, regulates lung development by regulating cell cycle and cell division related genes. Studies have shown that the decreased expression of Let-7c, a member of the Let-7 family, is closely related to the occurrence of multiple lung diseases (76). Previous studies have shown that lipoxin A4 (LXA4) can inhibit the development of BPD, but the mechanism is not clear. A study by Chen XQ et al., (77), suggested that the LXA4-imparted protective effects on hyperoxia-induced lung injury are mediated by upregulation of Let-7c and inhibition of transforming growth factor-β1 (TGF-β1) and subsequent downregulation of TGF-β1 signaling pathway.

In summary, there are 10 microRNA down-regulated, and three microRNA up-regulated. The main mechanisms of them involve the regulation of proliferation, migration, apoptosis, angiogenesis, inflammation and extracellular matrix, thus affecting the development of alveoli and blood vessels.

### LncRNA

Long non-coding RNA (LncRNA) is a class of RNA molecules with a length of more than 200 nt (32). Instead of encoding proteins, it regulates the expression level of gene in RNA level, like epigenetic regulation, transcriptional regulation, post transcriptional regulation, etc. A Recent study (78) have shown that LncRNA is involved in many important regulatory processes, such as chromosome silencing, genomic imprinting, chromatin modification, transcriptional activation, transcriptional interference, and intranuclear transport. These regulatory roles of LncRNA have also attracted extensive attention. Latest studies have shown that LncRNA has been involved the incidence and development of BPD, and the summary is as follows.

Metastasis associated lung adenocarcinoma transcript 1 (MALAT1) was found in metastasis tissues of patients with non-small cell lung cancer and were overexpressed. MALAT1 was closely related to pulmonary diseases (79). It has been reported that the expression of MALAT1 in lung tissue in WT mice is significantly lower than that in BPD mice. Meanwhile, the expression of MALAT1 in blood samples from preterm infants with BPD was significantly increased compared to normal infants. Knockdown of MALAT1 induced apoptosis in WI-38 cells, and the expression of CDC6 was down-regulated (80). A study by Yangi et al., (81), indicates that MALAT1 decreases the expression of miR-129-5p, promoting cell growth and inhibiting the development of bronchopulmonary dysplasia. Besides, MALAT1 increased the expression of high-mobility group protein 1 (HMGB1), which promote inflammation as the disease progressed. Moreover, lncRNA AK033210 is downregulated in BPD, which is associated with Tnc (82).

LncRNA H19 has been found to promote pulmonary fibrosis by regulating the miR-196a/ Collagen I alpha 1 (COL1A1) Axis (83). The researchers (84) find that lncRNA H19 expression is significantly increased in lung tissues of neonatal mice with hyperoxia-induced BPD. With the in-depth study, lncRNA H19 was knocked down and overexpressed in A549 cells, and MAPK signaling response and inflammatory factors were detected. In conclusion, lncRNA H19 has also been confirmed to regulate inflammatory responses via MAPK signaling pathway.

**Figure 1:**
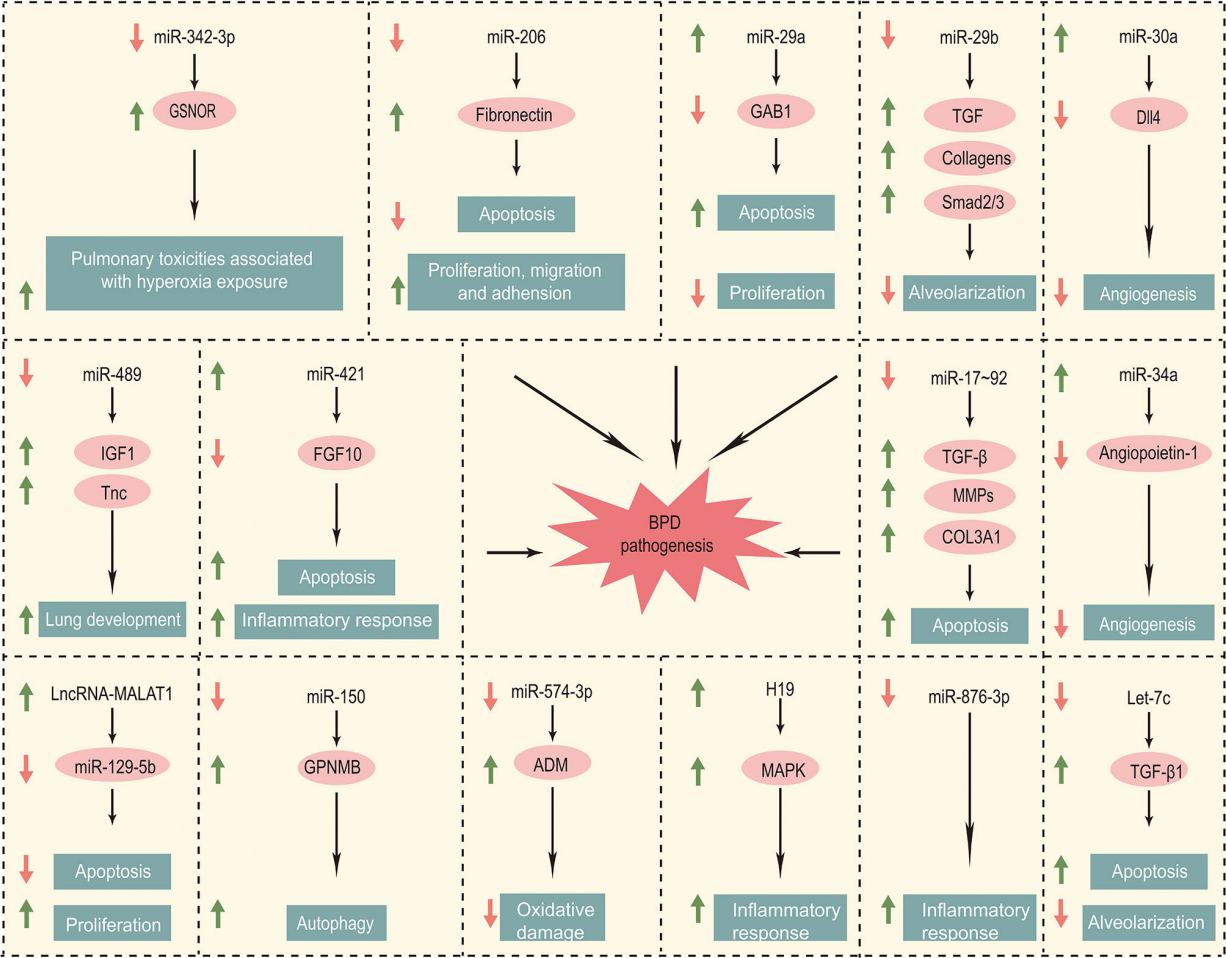
Non-coding RNA involved in the pathogenesis of BPD.

Non-coding RNAs currently implicated in bronchopulmonary dysplasia are summarized in the Table 1. The pathogenesis of BPD regulated by these non-coding RNAs is elaborated in the Figure 1.

**Table 1:** List of non-coding RNA currently implicated in bronchopulmonary dysplasia.

**Non-coding RNA**	**Mechanisms**	**Type of non-coding RNA**	**Expression**	**Cells and tissues**	**References**
miR-17~92	Targeting TGF-β, MMPs and COL3A1, decreased alveolarization	MicroRNA	Downregulated	BPD mouse lung tissues, Lung tissues of BPD neonates, plasma samples of BPD neonates	(44, 45)
miR-342-3p	Upregulation of GSNOR protein	MicroRNA	Downregulated	BPD mouse lung tissues	(46)
miR-206	Targeting fibronectin 1, inducing cell apoptosis, reducing cell proliferation, migration and adhesion	MicroRNA	Downregulated	BPD mouse lung tissues, plasma samples of BPD neonates	(54)
miR-29a	Targeting GAB1, inducing cell apoptosis, reducing cell proliferation	MicroRNA	Upregulated	BPD mouse lung tissues, bronchoalveolar stem cells	(55, 56)
miR-29b	Upregulation of TGF-β and Smad2/3, leading to ECM deposition, decreased alveolarization	MicroRNA	Downregulated	BPD mouse lung tissues, plasma samples of BPD neonates	(57)
MiR-30a	Targeting Dll4, downregulating angiogenesis	MicroRNA	Downregulated	BPD mouse lung tissues, plasma samples of BPD neonates	(60–62)
miR-34a	Targeting angiopoietin-1, downregulating angiogenesis	MicroRNA	Upregulated	BPD mouse lung tissues, type 2 alveolar epithelial cells in neonates with BPD	(63)
miR-489	Upregulation of IGF-1 and Tnc	MicroRNA	Downregulated	BPD mouse lung tissues, lung tissues of BPD neonates	(65)
miR-150	Targeting soluble GPNMB, inducing autophagy	MicroRNA	Downregulated	BPD mouse lung tissues	(68, 69)
miR-421	Targeting FGF10, promoting the apoptosis of MLE-12, aggravating the inflammatory response	MicroRNA	Upregulated	BPD mouse lung tissues, type II alveolar epithelial cell line MLE-12	(72)
MiR-574-3p	Targeting ADM, reducing oxidative damage	MicroRNA	Downregulated	BPD mouse lung tissues, plasma samples of BPD neonates	(73, 74)
miR-876-3p	aggravating the inflammatory response	MicroRNA	Downregulated	BPD mouse lung tissues, NHBE cells	(75)
Let-7c	Targeting TGF-β1, downregulation of TGF-β1 signaling pathway,inducing cell apoptosis, decreased alveolarization	MicroRNA	Downregulated	BPD mouse lung tissues, mouse lung epithelial cells (MLE-12 cells) and mouse embryonic fibroblasts (NIH/3T3 cells)	(77)
MALAT1	Targeting miR-129-5b, inhibiting cell apoptosis, promoting cell proliferation	Long non-coding RNA	Upregulated	WI-38 cells, A549 cells, BPD mouse lung tissues	(80, 81)
AK033210	Upregulation of Tnc	Long non-coding RNA	Downregulated	BPD mouse lung tissues	(82)
H19	Enhancing MAPK signaling pathway, inducing inflammatory responses	Long non-coding RNA	Upregulated	BPD mouse lung tissues	(84)

## Conclusions

BPD is one of the most serious lung diseases in preterm infants. So far, the pathogenesis of BPD is not yet clear, and there is no effective treatment. In this review, we summarize the non-coding RNA, including 13 microRNA and 3 lncRNA, associated with BPD. As for microRNA associated with BPD, the expression of miR-29a, miR-30a, miR-34a, miR-421 increased and the rest of microRNA decreased. LncRNA MALAT1 and H19 upregulated in BPD, while AK033210 downregulated in BPD. All the changes in microRNA and lncRNA related to BPD can be verified in mice lung tissues. MiR-17~92, miR-206, miR-29b, miR-30a, miR-489, and miR-547-3p can be validated in plasma samples or lung tissues of BPD neonates. As for mechanisms, miR-489 and AK033210 are associated with Tnc. MiR-206, miR-29a, and miR-421 promoted cell apoptosis, while miR-17~92, Let-7c, MALAT1 inhibited cell apoptosis. MiR-30a and miR-34a are associated with angiogenesis, while miR-29b, miR-421, miR-876-3p, and lncRNA H19 regulate inflammatory response. MiR-17~92, miR-29b, and Let-7c are associated with extracellular matrix formation and alveolarization. In summary, non-coding RNA is involved in the incidence and development of BPD by regulating the expression of genes related to proliferation, migration, apoptosis, angiogenesis, inflammation and extracellular matrix in alveolar epithelial cells and vascular endothelial cells.

At present, the clinical diagnosis and treatment of BPD is still lack of high sensitivity and specificity of detection indicators. More and more research results confirm that non-coding RNA plays an important role in the growth and development of lung tissue, so non-coding RNA can be used as a predictor or prognostic indicator of BPD, and also provide a new target for the treatment of BPD. Expression of specific non-coding RNAs are altered in BPD and their levels in the circulation often reflect the changes in expression of their lung-specific counterparts, exploiting these biomolecules as diagnostic tools seems an obvious goal. Some drugs, such as small interfering RNAs (siRNAs) or microRNA mimics, can be designed for regulating expression of microRNA, then promote angiogenesis and alveolar development, so as to achieve the purpose of treating BPD. We therefore believe that non-coding RNA is of great significance for the diagnosis and treatment of BPD.

## Author Contributions

YX: conception and design. YW: administrative support. Both authors: final approval of manuscript and manuscript writing.

## Conflict of Interest

The authors declare that the research was conducted in the absence of any commercial or financial relationships that could be construed as a potential conflict of interest.

## Publisher's Note

All claims expressed in this article are solely those of the authors and do not necessarily represent those of their affiliated organizations, or those of the publisher, the editors and the reviewers. Any product that may be evaluated in this article, or claim that may be made by its manufacturer, is not guaranteed or endorsed by the publisher.
